# Traumatic Hysteria

**Published:** 1901-09-28

**Authors:** 


### Traumatic Hysteria.

When as the result of an injury, or perhaps of that sudden shock to the nervous system which arises in the presence of imminent danger or profound terror, a person's mental characteristics are changed, and a condition is set up analogous with that which in women we call hysteria, it becomes a very serious question indeed how long the new state of affairs will last, and what are the probabilities of the patient ever again returning to that condition of mental stability in which he was before the occurrence. With the object of obtaining some data upon which to judge of the chances of persons so afflicted, Dr. Pearce Bailey,[1] of New York, describes the subsequent histories of a number of such cases in regard to which litigation had taken place. He says that it is easy to understand why hysteria is often misunderstood and the sufferer from it misjudged, for the disease, though essentially mental, finds its most conspicuous expression in physical symptoms, which are perplexingly similar to those caused by diseases of known material basis. The most important manifestations which these forms of hysteria cause are paralysis and defects in the special sense apparatus of sight, taste, smell, hearing, and common sensibility. These symptoms differ in mode of origin, in association and in character from those due to recognisable injury of the nervous system, and their origin would be sought for in vain either in the special sense organs or in their central connections or even in the specialised brain regions which have to do with the registration of sensorial impressions. The defect must be allocated to far higher spheres, to regions about which psychology may speculate, but about which pathological anatomy is dumb. Whatever may be the actual condition of the lower nervous planes in such cases their activities do not enter into the practical life of the individual. The belief of the patient in his own incapacity is absolute, and often so persistent as to deprive him of his usefulness for a considerable period of time. The author of this paper relates a series of such cases. In some a curiously rapid recovery seems to have taken place after compensation had been given, but in others this was not so and the disease persisted. Unfortunately, says Dr. Bailey, physicians generally believe that there is nothing the matter with hysterics, that they are posers and gallery-players, and that once they are paid for their fancied injuries, they will be as well as ever they were. In forming such opinions the fact seems to be lost sight of that a fixed idea may disorganise the life of an individual as much as a surgical injury, and that it may at any time take root so deep that it cannot be gotten rid of. Such a possibility then is present in every case of traumatic hysteria, and we have to consider what circumstances predispose to such permanence of the morbid state.

First of these is heredity. As in all nervous and mental diseases bad heredity throws an ominous shadow on prognosis, for instability of nervous function is the natural birthright of the children of those who are degenerate from any cause, and such persons are prone to develop mental anomalies from slight disturbances. On the other hand, the teaching of Charcot that hysteria never develops except in persons who are hereditarily or acquiredly degenerate is very difficult to substantiate. At any rate, it would seem that the majority of the victims of traumatic hysteria here related had as good family records as most people, and were quiet, orderly healthy citizens up to the occurrence of the accident. Some, on the other hand, had been hysterical before, and all the accident did was to intensify a pre-existing condition.

Then as to age. This is one of the most important considerations in regard to prognosis. The older the patient the greater is the danger of the condition being permanent. In young children the physical symptoms of hysteria almost always pass away in the course of a few weeks, while in persons under 35 years of age, if they are not seriously handicapped by heredity, recovery can be confidently looked for in the vast majority of cases.

Pre-existing chronic disease makes it more difficult to throw off hysteria, alcoholism and arterio-sclerosis being particularly unfavourable to recovery from functional nervous disorders. The fundamental cause of hysteria is to be found in the mental state, and it is the patient's purely psychological characteristics of attention, of memory, of mood, emotion and temperament, of will and of conduct, that are the most important considerations—not the mere extent or position of the physical symptoms. Finally, there is the question what we mean by recovery, and how far a man who has "recovered," as the saying is, either with or without compensation, is quite the same man as before. This is a matter which we think requires more serious and less flippant consideration than it sometimes receives. That a man recovers from the local manifestations is obvious enough, and the fact that after compensation he loses his paralysis, and can do his work, can use his hammer or his pen, and may straightaway marry and beget children, is often taken as showing the weakness of juries and the depravity of human nature. But is that man the same as before? Dr. Bailey says, "The exact definition of the word 'recovery' is in this sense hard to give, but it is diametrically opposed to the teachings of psychology to suppose that a fixed idea which has persisted for several months or years can vanish immediately without leaving trace. The paralysis, or blindness, or anæsthesia may disappear like magic. But I cannot believe but that there is left an unstable nervous state which will persist for at least as long as the more serious symptoms persisted," an instability which may not interfere with working capacity, but must interfere with the comfort of the patient to a considerable degree. Further, it is worth while to remember that "it is practically impossible for anyone to get well from this disease as long as money questions are at stake, and that the longer such questions are undecided the more the symptoms tend to permanency and the less probable it becomes that they will ever disappear."

[1] New York Med. Record, Aug. 24.

